# Findings on the Central Auditory Functions of Endemic Disease Control Agents

**DOI:** 10.3390/ijerph18137051

**Published:** 2021-07-01

**Authors:** Patrícia Arruda de Souza Alcarás, Bianca Simone Zeigelboim, Maria Cristina Alves Corazza, Débora Lüders, Jair Mendes Marques, Adriana Bender Moreira de Lacerda

**Affiliations:** 1 Audiology Department, Speech Therapy School, University of Western São Paulo, UNOESTE, Presidente Prudente 19050-920, SP, Brazil; patricialcaras@hotmail.com (P.A.d.S.A.); corazza@unoeste.br (M.C.A.C.); 2 Post-Graduate Program in Communication Disorders, Tuiuti University of Paraná, UTP, Curitiba 82010-330, PR, Brazil; bianca.zeigelboim@utp.br (B.S.Z.); debora.luders@utp.br (D.L.); jair.marques@utp.br (J.M.M.); 3 Audiology Department, Speech Language and Audiology School, Medicine Faculty, Université de Montréal, UdeM, Montreal H3C 3J7, QC, Canada

**Keywords:** community health agents, pesticides, hearing, hearing loss, neurotoxicity

## Abstract

This study aimed to assess the central auditory functions of endemic disease control agents. This cross-sectional cohort study comprised two groups: the exposed group, with 38 male endemic disease control agents with simultaneous occupational noise and pesticide exposure; and the control group, with 18 age- and sex-matched workers without occupational noise and/or pesticide exposure. All participants underwent pure-tone audiometry, brainstem auditory evoked potentials, dichotic digits test, and transient-evoked otoacoustic emissions suppression effect. There was a significant inter-group difference in waves III and V absolute latencies, and interpeak I–III and I–V latencies bilaterally, with worse results found in the exposed group. Abnormal dichotic digits test results occurred more often in the exposed group, with a significant association between pesticide- and noise-exposure and the abnormal results (*p* = 0.0099). The transient-evoked otoacoustic emissions with suppression effect did not yield significant inter-group differences. It was concluded that pesticide and noise exposure induce harmful effects on the central auditory functions, particularly on the brainstem and figure-ground speech-sound auditory skills.

## 1. Introduction

Endemic disease control agents are professionals who work in direct contact with pesticides, from preparing to applying the pesticides to infested environments [1,2]. Their health is thus highly at risk, as pesticides are chemical substances that can be absorbed through the skin, ingestion, and inhalation [3,4].

Besides pesticide exposure, endemic disease control agents are also exposed to noise generated by automatic backpack sprayers and truck-mounted ultra-low volume (ULV) sprayers they work with. Therefore, it has been recommended that sound pressure level generated from the automatic equipment should be measured in studies that involve pesticide- and noise-exposed groups [5].

Pesticides have various effects on the auditory system. Authors have reported that insecticides and organophosphate pesticides can disturb the efferent auditory system. This phenomenon is caused by the inhibition of acetylcholinesterase, which in turn leads to the accumulation of acetylcholine in the peripheral and central auditory pathways [6,7,8,9]. Consequently, the action potential of the efferent system from the superior olivary nucleus to the cochlea is disrupted [8,10,11].

Studies in humans reveal that pesticides, either alone or in combination with noise, damage not only the peripheral auditory system [12,13,14,15], but also the central auditory system [8,16,17,18,19,20,21,22,23,24,25].

A study assessed the central auditory system of endemic disease control agents who were exposed to pyrethroid and organophosphate insecticides. Their results showed that 56% of endemic disease control agents had a central auditory dysfunction, with a relative risk of central auditory dysfunction reaching 7.58 [16]. Similar results were observed in other studies with farmworkers exposed to organophosphate pesticides [19], herbicides, insecticides, and fungicides [22]. Using long-latency potentials (P300), the authors demonstrated an increase in the P300 wave latency of farmworkers exposed to organophosphate insecticides [18]. Such results suggest that chronic pesticide exposure can cause delays in the neurophysiological processes and alter the central auditory system. Similar results were observed in a study involving 14 workers exposed to organophosphate insecticides [25].

When assessing the central auditory functions of tobacco growers exposed to organophosphate insecticides, a study found an alteration in the dichotic digits test (DDT) and random gap detection test (RGDT) [8]. They concluded that these workers showed signs of central auditory dysfunction caused by a decrease in temporal processing and binaural integration skills. Besides the harmful effects caused by the organophosphate pesticides on the central auditory pathways, studies also show similar effects on the brainstem auditory pathways [9,20,23,24] and the efferent auditory system, two systems that control the active process of the outer cochlear hair cells [21,26,27].

The abovementioned studies highlighted the possible alterations in the central auditory system of pesticide-exposed populations especially in the agricultural sector. However, few studies demonstrated the effects of pesticides on the brainstem auditory pathways [16] and the medial olivocochlear efferent system [26] in the public health sector.

It should be noted that organophosphate insecticides, when combined with noise, may have an enhanced deleterious effet on the auditory system [12,28]. Occupational noise exposure often causes progressive, irreversible, sensorineural, usually binaural hearing loss in exposed subjects [29]. A study with insect-pest controllers conducted a quantitative assessment of noise levels and tried reducing the level of noise exposure by modifying the equipment. The equivalent noise level measured was 98.5 dB(A), which corresponded to a dosage of 321.6% at the time of the assessment. The study concluded that even when wearing earmuffs—considering a mean theoretical noise reduction rating of 25 dB—there was still a residual noise exposure of 86.3 dB(A) [30].

Given the size of the population and their high risk of exposure to toxic chemicals that could be comparable to that of farmworkers [31], this study aimed to assess the central auditory functions of endemic disease control agents simultaneously exposed to pesticides and noise.

## 2. Materials and Methods

This is a cross-sectional cohort study, approved by the Research Ethics Committee via *Plataforma Brasil* under certificate number CAAE 48572415.8.0000.5225 and evaluation report 1.242.014. It was developed at the speech-language-hearing teaching clinic of a private university in Paraná, Brazil, with civil servants of the state of Paraná. The study was conducted in partnership with the Syndicate of Federal Civil Servants in Health, Labor, Social Security, and Welfare of the State of Paraná (SindPrevs/PR), Federal University of Paraná, Paraná State Department of Health, and the Public Ministry of Labor.

The present study is an integral part of a doctoral thesis aiming to determine the impact of simultaneous exposure to pesticides used in the public health sector and to noise on the peripheral and central auditory, and on the vestibular systems of endemic disease control agents. A questionnaire was used to gather information on the health and working conditions of the participants, along with an informal interview about their work and a battery of audiological tests. Part of the information presented in this study was taken from that questionnaire, while another part came from the informal interview conducted during the audiological assessment. Moreover, the Syndicate of Civil Servants of the State of Paraná furnished information on noise levels and preventive actions at work. Thus, the information presented in this study was not collected with a single instrument and therefore we reckoned the questionnaire did not need to be included.

The inclusion criteria for the exposed group were being an endemic disease control agent, being a civil servant of the State of Paraná, and being over 18 years old. The exclusion criterion was having mixed or conductive hearing loss. The participants were recruited by oral invitation from the person in charge of the SindPrevs/PR. To those who were interested, the union offered transportation to the site of the field study.

The control group included participants who were age- and sex- matched to the exposed group. The exclusion criteria were having an occupational history of exposure to physical and chemical agents, and having conductive and/or mixed hearing loss. Recruitment was by way of an invitation letter from the researchers. Upon agreeing to participate and signing the informed consent form, a total of 56 workers were included in the sample of the study. They were divided into two groups, namely: the exposed group (EG) and the control group (CG). The EG included 38 male endemic disease control agents, aged 48 to 72 years (56.1 ± 5.8), with more than 20 years (31.2 ± 3.8) of occupational exposure to both insecticides (organophosphates and pyrethroids) and noise (caused by automatic backpack sprayer and/or truck-mounted heavy ULV sprayer). Data provided by the SindPrevs/PR showed that the time-weighted averages (TWA) of noise generated by the automatic backpack sprayers were 107 dBA. The truck-mounted heavy ULV sprayer generated noise equivalent to 75 dBA inside the vehicle with shut windows and 110 dBA outside the vehicle. Working hours with the heavy ULV normally extended from five to eight o’clock in the morning, and possibly to ten o’clock in the morning—i.e., working activities in the morning could last from three to five hours. In the afternoon, work started at four and ended at eight o’clock, possibly extending to ten o’clock at night. The total working hours during the afternoon could total from four to six hours. The overall total mean, excluding the commute, ranged from six to ten hours of daily activity with the heavy ULV. As for the backpack sprayers, the daily working hours added up to eight hours.

Besides spraying the insecticides, the exposed participants reported that their professional activity involved preparing, diluting, storing the substances, and cleaning the material used. Of the 38 endemic disease control agents, 34 (89.5%) reported wearing personal protective equipment (PPE), such as breathing masks, disposable clothes, hats, boots, waterproof gloves, and protective goggles. Twenty-nine workers (76.3%) reported wearing earplugs when exposed to noisy equipement, though this information could not be verified. The CG comprised 18 male workers, aged 48 to 70 years (mean = 56 years; SD = 5.6). Their professional occupation included administration, nursing, commerce, business, law, teaching, cartography, tax audit, and health surveillance.

All the participants were submitted to inspection of the external acoustic meatus, pure-tone audiometry, acoustic immittance, brainstem auditory evoked potentials (BAEP), dichotic digits test (DDT) assessment—binaural integration stage [32], and assessment of the transient evoked otoacoustic emissions (TEOAE) suppression effect [33] (Table 1).

The results obtained from each group underwent descriptive statistical analysis with mean, standard deviation, and minimum and maximum values.

The BAEP was analyzed between participants with high-frequency pure-tone auditory threshold up to 25 dB HL, and between participants with high-frequency pure-tone auditory threshold from 30 to 50 dB HL, to assess whether high-frequency hearing loss influenced the BAEP findings.

Student’s *t*-test was used to compare the means of BAEP, hearing thresholds at each frequency, and overall response of the OAE suppression effect, between EG and CG. This test is appropriate to compare the means of two small samples, as long as such samples come from populations with a normal probability distribution. This condition was verified with the Shapiro–Wilk method before applying the *t*-test, validating that such a prerequisite was met. The ANOVA test was used to analyze the results of the DDT between the groups—likewise, verifying the necessary prerequisites before applying it (same population variances and normality of the samples). The chi-square test with Yates’ correction for continuity was used to determine the correlation between the number of altered cases in the DDT and risk exposure. Pearson correlation was used to determine the association between the participants’ age and the results in the DDT. The 0.05 (5%) significance level was used [34].

## 3. Results

**Figure 1:**
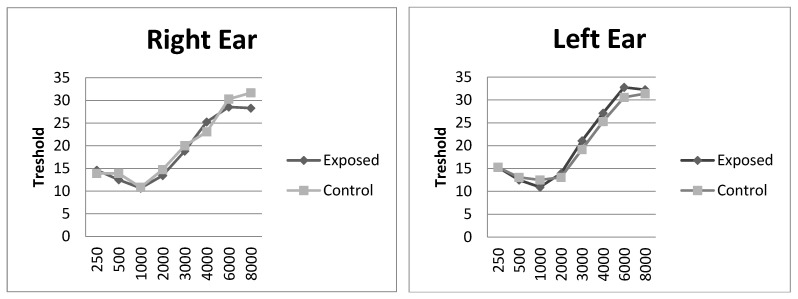
Mean auditory thresholds of the participants of the study—exposed group (N = 38) and control group (N = 18).

The EG and CG participants’ mean auditory thresholds per ear (right and left) are shown in Figure 1.

**Figure 2:**
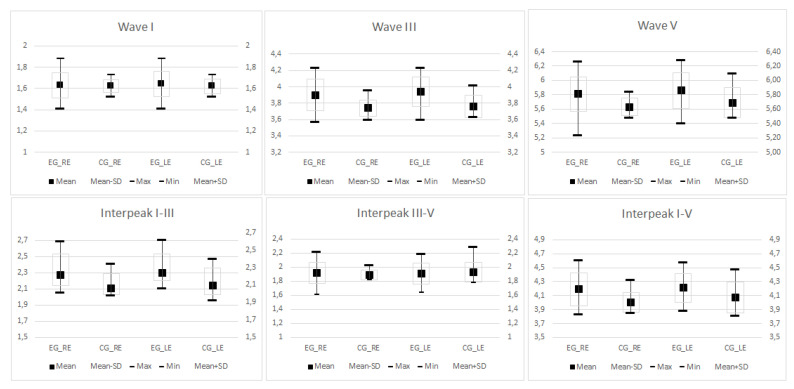
Boxplots of the mean, standard deviation, and maximum and minimum values of the absolute and interpeak latencies of the BAEP, obtained in the right (RE) and left (LE) ears of the exposed (EG) and control (CG) groups, with pure-tone auditory threshold up to 25 dB HL at the frequencies from 2000 to 4000 Hz.

The means and standard deviations of the absolute latencies (I, III, and V), interpeak latencies (I–III, III–V, and I–V), and amplitude of waves I and V of participants with pure-tone auditory threshold up to 25 dB HL are shown in Table 2 and Figure 2, according to right and left ear. There was a significant statistical difference between the groups in the latencies of waves III and V, and interpeak latencies I–III and I–V of both ears. The EG had longer wave and interpeak latencies compared to those of the CG. Such a difference was not observed in the latency of wave I, interpeak interval III–V, or amplitude of waves I’ and V’ (Table 2).

The BAEP findings comparing the participants with pure-tone thresholds from 30 to 50 dB HL are shown in Table 3. A total of 12 right and 13 left ears of the EG and six right and six left ears of the CG were analyzed. There was no statistical difference in the BAEP recordings between the groups (with auditory thresholds from 30 to 50 dB HL) (Table 3).

Of the 38 endemic disease control agents, 35 performed the DDT—binaural integration stage; three were excluded because of the equipment’s unavailibility at the time of their assessment. As for the CG, all the participants performed the DDT. Only participants with four-frequency mean up to 25 dB HL at the frequencies of 500 Hz, 1000 Hz, 2000 Hz, and 4000 Hz were included in the DDT analyses. Hence, the EG comprised 30 participants, and the CG comprised 14 participants.

**Figure 3:**
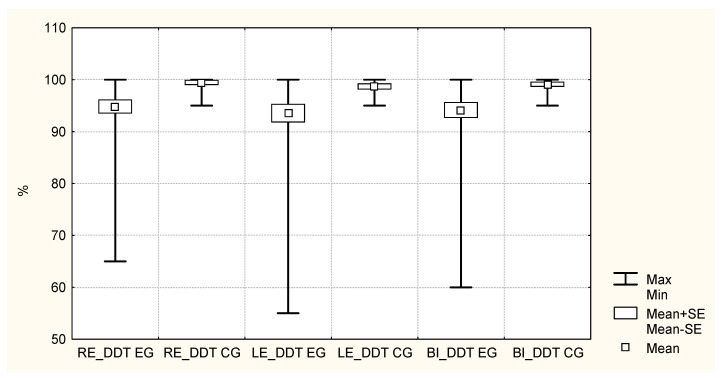
Boxplot of the exposed group (EG) and control group (CG) participants’ scores in the dichotic digits test (DDT) of the right ear (RE), left ear (LE), and binaural (BI).

The boxplot (mean, standard deviation, minimum, and maximum values) for the right ear, left ear, and binaural DDT results are shown in Figure 3. There is great variation in DDT results in the EG, which was not observed in the CG.

In the DDT, there were 13 abnormal (43.3%) and 17 normal results (56.7%) in the EG, whereas the CG (n = 14) all had normal results (100%). The chi-square test with Yates’ correction for continuity resulted in *p* = 0.0099 (*p* < 0.01)—i.e., the result revealed a significant association between pesticide and noise exposure and abnormal results. The results in the Pearson correlation suggested an association between age and DDT results (RE DDT, LE DDT, and binaural DDT) and between the right ear, left ear, and binaural thresholds (for the frequencies of 0.25, 0.5, 1, 2, 3, 4, 6, and 8 kHz), as seen in Table 4.

The Pearson correlation coefficient revealed no significant correlation between age and the four-frequency mean of auditory thresholds with the RE DDT, LE DDT, and binaural DDT scores. Therefore, age and auditory thresholds did not influence the DDT results.

The DDT results are shown in Table 5 and were not adjusted for age and auditory threshold, as they did not have significant correlations.

The DDT scores of the EG were compared with those of the CG with the ANOVA test. The RE DDT resulted in the statistic F = 5.68 (*p* = 0.0218); the LE DDT, in F = 4.23 (*p* = 0.0460); and the binaural DDT, in F = 5.69 (*p* = 0.0216). Thus, the difference in the means of the DDT results between both groups was significant (*p* < 0.05) in the three cases, with worse results in the EG.

The TEOAE suppression effect was assessed in the participants who passed the TEOAE (EG = 14; CG = 12). In the EG, two subjects did not participate in the suppression effect assessment because the equipment was not available when they were examined. The mean, minimum and maximum values, and standard deviation of each frequency band (1, 1.4, 2, 2.8, and 4 kHz), as well as the general response of the TEOAE suppression effect by ear (right and left), are shown in Table 6. The *t*-test revealed no statistically significant difference in the frequencies analyzed and in the general response between the groups in either the right or left ear.

## 4. Discussion

This study examined the effects of simultaneous noise and pesticide exposure—particularly to organophosphate and pyrethroid insecticides—on the central auditory functions of endemic disease control agents.

In 2019, the Brazilian Ministry of Health published a report on the various health risks and protection measures for endemic disease control agents responsible for spraying pesticides. Regarding hearing, the report highlights the noise levels of the equipment they use and recommends measures to reduce the noise exposure. The same report describes some of the risks of chemical exposure, including neurological disorders, with no specific mention of hearing [35].

BAEP consists of an early auditory potential that indicates the integrity of the afferent auditory pathway up to the brainstem with sound stimuli. The waves generated in the auditory pathways help identify lesions in the upper and lower brainstem. Waves I and II are generated from the cochlear nerve, while wave III potential begins at the cochlear nuclei, and waves IV and V begin at the lateral lemniscus. The interpeak of waves I–III reflects the sound conduction between the auditory nerve and the projections of the cochlear nuclei. The interpeak of waves III–V is generated in the brainstem, between the cochlear nuclei and the upper lateral lemniscus [36].

In this study, there was an increase in waves III and V absolute latencies, and I–III and I–V interpeak latencies in the EG when compared to the CG with auditory thresholds up to 25 dB HL at the frequencies from 2000 to 4000 Hz [36]. This suggests a neurotoxic effect on the brainstem in the EG, affecting the synapses of neurons located between the cochlear nuclei and the endings in the lateral lemniscus [36,37].

The BAEP findings in this study corroborate other studies that showed a deleterious effect of pesticides on the auditory system [9,23]. A study conducted in 2012 aimed to determine the integrity of the peripheral and central auditory pathways in patients exposed to organophosphate compounds. Their results showed that waves I, III, and V were prolonged [23]. In another study conducted in 2018, such an increase in wave latencies was observed in the absolute latency of wave V in both ears [9]. However, in other studies, there was no difference in the BAEP recordings in pesticide-exposed in comparison with nonexposed populations [20,24].

For the DDT, the conventional pure-tone thresholds were initially compared between the participants whose four-frequency mean was up to 25 dB HL—which is the inclusion criterion for DDT analyses. The result did not reveal a significant difference between the groups (*p* < 0.05). Similar findings were observed in another study [8]. The difference in the number of normal and abnormal results revealed a significant association between pesticide and noise exposure and the abnormal results. Similar results were observed in a study conducted in 2017, in which a group of tobacco growers presented worse DDT performance than a CG [8]. There were similar findings in a study with a solvent-exposed population [38]. When performances of the right and left ears were analyzed, the results revealed a significant difference between the groups in the comparisons between right ear, left ear, and binaural. Furthermore, when assessing the results of the binaural test, the mean DDT result was always worse in the exposed as compared to the nonexposed population. The same result was observed in another study with a pesticide-exposed population [8].

A single test is not enough to characterize alteration in the central auditory processing. However, the DDT—binaural integration stage assessed the figure-ground speech-sound auditory skill. Performances were worse in the group of pesticide- and noise-exposed workers. Further studies should be carried out with a more comprehensive battery of tests to better determine the influence of the pesticides on central auditory processing.

Regarding the assessment of the TEOAE suppression effect, the findings revealed no difference between the groups at the specific frequencies of 1000, 1400, 2000, 2800, and 4000 Hz, nor in the general response. The findings are consistent with other studies, which similarly found no difference between the exposed and nonexposed to risk agents [39,40]. Nevertheless, in other studies [21,26,27], there was a significant difference in the OAE suppression effect in a pesticide-exposed population in comparison with a CG. In those studies [21,26,27], however, the population had a lower mean age (under 40 years) compared to the present study (mean = 56 years).

The increase in age reduces the suppression effect [41]. When studying the changes in the efferent system with advancing age in a population ranging from 10 to 80 years old, a significant decrease was observed in the 61- to 70-year-old participants. When investigating the effect of age on the medial olivocochlear pathway, with the analysis of the amplitude of the otoacoustic emissions with contralateral stimulation in 75 individuals grouped according to their age (20 to 30 years, 30 to 40 years, 40 to 50 years, 50 to 60 years, and over 60 years), the authors observed a decrease in suppression beginning at 40 years. They concluded that aging impairs the effectiveness of the medial olivocochlear pathway [42]. Thus, the authors reported that the reduction in suppression effect with advancing age is due to the natural degeneration process of the involved structure in the efferent pathway. Such is the case for the medial olivocochlear pathway, as part of its axon myelin sheath deteriorates over the years [42].

### 4.1. Recommendations Based on the Results of This Study

Based on the results presented in this study, assessment of the central auditory functions is recommended for populations using pesticides, either alone or in combination with noise. Such is the case for endemic disease control agents, who are exposed to these ear-damaging agents while working in a public health setting. In particular, it is recommended that brainstem auditory evoked potentials and the dichotic digits test be conducted on these workers.

### 4.2. Limitations of the Study

An important limitation of this study is the age of its population. In advanced age, hearing decreases due to the natural degeneration process of the structures involved in the afferent, central, and efferent auditory pathways. No objective measurements were taken to evaluate the participants’ exposure to pesticides, and we were unable to obtain more precise information on their history of exposure to risk agents. This study did not provide specific contributions regarding noise exposure apart from that of pesticides. As mentioned in the methodology, a single questionnaire was not used to collect information on working conditions and health. The education and socioeconomic levels were not equivalent between participants, which could explain some of the differences in the test results. Lastly, this study has a limitation common to all cross-sectional ones, as it was not possible to conclude a causal relationship, but only an association between the pesticide exposure and central auditory alterations in the studied population.

## 5. Conclusions

These results lead to the conclusion that pesticide and noise exposure are associated with harmful effects on the central auditory functions, particularly on the brainstem and figure-ground speech-sound auditory skills, as identified with BAEP and DDT.

## Figures and Tables

**Table 1:** Description, recording parameters, and analysis criteria of auditory tests performed in the exposed group and control group.

Test	Sample Included	Recording Parameters	Analysis Criteria
BAEP	Participants with auditory thresholds up to 25 dB HL at the frequencies from 2000 to 4000 Hz; Participants with auditory thresholds from 30 to 50 dB HL at the frequencies from 2000 to 4000 Hz.	Equipment: *Vivosonic/Integrity^®^, V500, Vivosonic Inc, Toronto, ON, Canada* Surface electrodes: forehead (Fz—positive electrode), and right and left mastoids (M1 and M2—negative electrodes) Transducer: E3-A insert earphone Immittance: <5 kOhms Promediations: 2000 stimuli Type of stimulus: click Presentation rate: 27.7 clicks/s Filters: 100–3000 Hz Recording window: 12 ms Polarity: rarefied Intensity: 80 dBnHL	Presence of waves I, III, and V. Latency of waves I, III, and V. Interpeak latencies of waves I–V, I–III, and III–V. Amplitude of waves I’ and V’. The analysis criterion was the comparison between the records of the two groups.
DDT—binaural integration	Participants with a four-frequency mean (500, 1 kHz, 2 kHz, and 4 kHz) up to 25 dB HL.	Equipment: *Madsen^®^/Itera II, GN Otometrics, Ballerup, Denmark*. Additionnal material: CD by Pereira and Schochat (1997) [32], volume 2, track 3, and test 6. Intensity: 50 dB SL List: 80 digits	Up to 59 years old = score higher than 95%. 60 years or older, without hearing loss = 78%.
TEOAE	Participants with the presence of TEOAE and type A tympanometric curve.	Equipment: *Otodynamics^®^/ILO 292/V6, Hatfield, UK* Intensity of the stimulus: 60–65 dB SPL Intensity of contralateral white noise: 60 dB SPL Type of stimulus: linear click Number of stimuli: 200 sweeps with noise, and 200 sweeps without noise.	Comparison between the OAE results with and without contralateral noise in each frequency and general response. Minimum decrease in the general response of 0.5 to 1.0 dB.

**Table 2:** Mean and standard deviation of absolute latencies, interpeak latencies, and amplitudes of waves I’ and V’ obtained from the right and left ears of the exposed and control groups, with auditory thresholds of 25 dB HL at the frequencies from 2000 to 4000 Hz.

BAEP			Mean	SD	*p*-Value
Wave I	RE	exposed (N = 20)	1.63	0.12	0.3953
		control (N = 12)	1.62	0.06	
	LE	exposed (N = 18)	1.64	0.12	0.3579
		control (N = 12)	1.62	0.07	
Wave III	RE	exposed (N = 20)	3.90	0.19	0.0058 *
		control (N = 12)	3.74	0.10	
	LE	exposed (N = 18)	3.94	0.18	0.0034 *
		control (N = 12)	3.76	0.14	
Wave V	RE	exposed (N = 20)	5.81	0.24	0.0111 *
		control (N = 12)	5.63	0.12	
	LE	exposed (N = 18)	5.86	0.25	0.0312 *
		control (N = 12)	5.69	0.21	
Interpeak I–III	RE	exposed (N = 20)	2.27	0.18	0.0053 *
		control (N = 12)	2.11	0.12	
	LE	exposed (N = 18)	2.30	0.15	0.0039 *
		control (N = 12)	2.14	0.15	
Interpeak III–V	RE	exposed (N = 20)	1.92	0.15	0.2608
		control (N = 12)	1.89	0.07	
	LE	exposed (N = 18)	1.91	0.15	0.3581
		control (N = 12)	1.93	0.14	
Interpeak I–V	RE	exposed (N = 20)	4.19	0.24	0.0093 *
		control (N = 12)	4.0	0.14	
	LE	exposed (N = 18)	4.21	0.21	0.0451 *
		control (N = 12)	4.07	0.22	
Amplitude I’	RE	exposed (N = 20)	0.14	0.10	0.0591
		control (N = 12)	0.09	0.05	
	LE	exposed (N = 18)	0.13	0.07	0.2374
		control (N = 12)	0.11	0.08	
Amplitude V’	RE	exposed (N = 20)	0.26	0.12	0.3207
		control (N = 12)	0.28	0.11	
	LE	exposed (N = 18)	0.30	0.09	0.2398
		control (N = 12)	0.27	0.14	

*t*-test at the 0.05 significance level (significant *p*-value *). Legend: RE = right ear; LE = left ear; SD = standard deviation.

**Table 3:** Mean and standard deviation of the absolute latencies, interpeak latencies, and amplitudes of waves I’ and V’ obtained in the right and left ears of the exposed and control groups, with auditory thresholds from 30 to 50 dB HL at the frequencies from 2000 to 4000 Hz.

BAEP			Mean	SD	*p*-Value
Wave I	RE	exposed (N = 12)	1.68	0.12	0.0830
		control (N = 06)	1.60	0.09	
	LE	exposed (N = 13)	1.64	0.11	0.0914
		control (N = 06)	1.57	0.08	
Wave III	RE	exposed (N = 12)	3.86	0.20	0.2898
		control (N = 06)	3.81	0.11	
	LE	exposed (N = 13)	3.87	0.19	0.0748
		control (N = 06)	3.75	0.04	
Wave V	RE	exposed (N = 12)	5.83	0.38	0.2372
		control (N = 06)	5.71	0.16	
	LE	exposed (N = 13)	5.78	0.31	0.5000
		control (N = 06)	5.78	0.12	
Interpeak I–III	RE	exposed (N = 12)	2.18	0.20	0.3723
		control (N = 06)	2.21	0.13	
	LE	exposed (N = 13)	2.22	0.17	0.2936
		control (N = 06)	2.18	0.06	
Interpeak III–V	RE	exposed (N = 12)	1.97	0.24	0.2718
		control (N = 06)	1.90	0.19	
	LE	exposed (N = 13)	1.91	0.25	0.1579
		control (N = 06)	2.02	0.09	
Interpeak I–V	RE	exposed (N = 12)	4.15	0.39	0.4053
		control (N = 06)	4.11	0.10	
	LE	exposed (N = 13)	4.14	0.25	0.2874
		control (N = 06)	4.20	0.06	
Amplitude I’	RE	exposed (N = 12)	0.08	0.05	0.2177
		control (N = 06)	0.10	0.05	
	LE	exposed (N = 13)	0.13	0.06	0.1516
		control (N = 06)	0.10	0.05	
Amplitude V’	RE	exposed (N = 12)	0.22	0.11	0.3595
		control (N = 06)	0.20	0.08	
	LE	exposed (N = 13)	0.23	0.12	0.1335
		control (N = 06)	0.17	0.06	

*t*-test at the 0.05 significance level (significant *p*-value *). Legend: RE = right ear; LE = left ear; SD = standard deviation.

**Table 4:** Pearson correlation of age and threshold with the right ear, left ear, and binaural dichotic digits test.

Correlation between	Correlation Coefficient (r)	*p*
Age and RE DDT	−0.2306	0.2201
Age and LE DDT	−0.0260	0.8914
Age and binaural DDT	−0.1306	0.4917
RE threshold and RE DDT	−0.2724	0.0736
LE threshold and LE DDT	−0.1016	0.5115
Binaural threshold and binaural DDT	−0.2324	0.1289

Legend: DDT = dichotic digits test; RE = right ear; LE = left ear.

**Table 5:** Dichotic digits test results for the exposed and control groups.

TEST	Exposed			Control		
	Mean	SD	95% CI	Mean	SD	95% CI
RE DDT	94.5	1.4	91.76–97.28	99.46	0.39	98.70–100.00
LE DDT	93.9	1.6	90.67–97.07	98.75	0.51	97.75–99.75
BI DDT	94.2	1.4	91.43–96.95	99.11	0.40	98.32–99.90

Legend: CI = confidence interval; SD = standard deviation; DDT = dichotic digits test; RE = right ear; LE = left ear; BI = binaural.

**Table 6:** Descriptive statistics of the TEOAE suppression effect at the frequencies of 1 kHz, 1.4 kHz, 2 kHz, 2.8 kHz, and 4 kHz, and general response of the exposed and control groups per ear.

	RIGHT EAR								
	Exposed (N = 14)				Control (N = 12)				
	Mean	SD	Min	Max	Mean	SD	Min	Max	*p*-Value
1 kHz	−0.44	2.66	−5.5	4.1	0.70	5.04	−11.3	6.6	0.2342
1.4 kHz	1.11	3.65	−8.7	4.6	0.07	2.75	−4	3.4	0.2133
2 kHz	−0.86	2.87	−5.4	4.5	−0.06	2.08	−4.1	2.8	0.2155
2.8 kHz	1.96	1.97	−0.7	6	1.26	3.12	−3.7	7.4	0.2470
4 kHz	0.33	2.10	−2.2	4.5	0.38	2.01	−3.2	4.4	0.4756
GenRes	0.15	−0.9	0.7	0.41	0.47	−0.7	2	0.72	0.0844
	**LEFT EAR**								
	**Exposed (N = 13)**				**Control (N = 14)**				
	**Mean**	**SD**	**Min**	**Max**	**Mean**	**SD**	**Min**	**Max**	***p*-Value**
1 kHz	−0.2	4.51	−6.5	7.1	0.70	5.50	−11	9.4	0.3238
1.4 kHz	0.0	2.33	−4	5.3	0.00	2.24	−3	4.1	0.5000
2 kHz	0.8	1.85	−1.8	4.4	−0.19	2.66	−4.7	5.4	0.1380
2.8 kHz	−0.4	2.66	−7.1	3.3	1.01	2.36	−5	4.5	0.0845
4 kHz	0.9	2.13	−1.7	6.8	0.31	2.39	−3.6	5.6	0.2560
GenRes	0.1	−1.6	1.5	0.85	0.46	−1.3	3.5	1.05	0.1696

*t*-test at the 0.05 significance level. Legend: RE = right ear; LE = left ear; N = number of participants; Min = minimum; Max = maximum; SD = standard deviation; GenRes = general response.

## References

[1] Carneiro F.F., Rigotto R.M., Augusto L.G.S., Friedrich K., Búrigo A.C. (2015). Dossiê ABRASCO: Um Alerta Sobre os Impactos dos Agrotóxicos na Saúde.

[2] Torres R. (2009). Agente de combate a endemias. Rev. Poli–Saúdeeduc. E Trab..

[3] Lima E.P., Lopes S.M.B., Amorin M.I.M., Araújo L.H.S., Neves K.T., Maia E.R. (2009). Pesticide exposure and its repercussion in the health of sanitary agents in the State of Ceará, Brazil. Ciência Saúde Coletiva.

[4] OSHA (2009). Occupational Safety and Health Administration. https://www.osha.gov/news/newsreleases/infodate-y/2018.

[5] Morata T.C., Little M.B. (2002). Suggested guidelines for studying the combined effects of occupational exposure to noise and chemicals on hearing. Noise Health.

[6] Koelle G.B. (1994). Pharmacology of organophosphates. J. Appl. Toxicol..

[7] Sidell F.R. (1994). Clinical effects of organophosphorus cholinesterase inhibitors. J. Appl. Toxicol..

[8] França D.M.V., Lacerda A.B.M., Lobato D., Ribas A., Dias K.Z., Leroux T., Fuente A. (2017). Adverse effects of pesticides on central auditory functions in tobacco growers. Int. J. Audiol..

[9] Singh M., Minhas R.S., Machhan P., Azad R.K., Mohindroo S. (2018). Audiological assessment in organophosphorus poisoning. Int. J. Otorhinolaryngol. Head Neck Surg..

[10] Cáceres T., Megharaj M., Venkateswarlu K., Sethunathan Nethunathan N., Naidu R. (2010). Fenamiphos and related organophosphorus pesticides: Environmental fate and toxicology. Rev. Environ. Contam. Toxicol..

[11] Secretaria de Estado da Saúde do Paraná–SESA/PR (2013). Protocolo de Avaliação das Intoxicações Crônicas por Agrotóxicos.

[12] Teixeira C.F., Augusto L.G.S., Morata T.C. (2003). Saúde auditiva de trabalhadores expostos a ruído e inseticidas. Rev. Saúde Pública.

[13] Körbes D., Silveira A.F., Hyppolito M.A., Murano G. (2010). Alterações no sistema vestibulococlear decorrentes da exposição ao agrotóxico: Uma revisão de literatura. Rev. Soc. Bras. Fonoaudiol..

[14] Kós M.I., Hoshino A.C., Asmus C.I.F., Mendonça R., Meyer A. (2013). Efeitos da exposição a agrotóxicos sobre o sistema auditivo periférico e central: Uma revisão sistemática. Cad. Saúde Pública.

[15] Gatto M.P., Fioretti M., Fabrizi G., Gherardi M., Strafella E., Santarelli L. (2014). Effect of potential neurotoxic on hearing loss: A review. Neurotoxicology.

[16] Teixeira C.F., Augusto L.G.S., Morata T.C. (2002). Occupational exposure to insecticides and their effects on the auditory system. Noise Health.

[17] Lizardi P.S., O’Rourke M.K., Morris R.J. (2008). The effects of organophosphate pesticide exposure on Hispanic children’s cognitive and behavioral functioning. J. Pediatr. Psychol..

[18] Dassanayake T., Gawarammana I.B., Weerasinghe V., Dissanayake P.S., Pragaash S., Dawson A., Senanayake N. (2009). Auditory event-related potential changes in chronic occupational exposure to organophosphate pesticides. Clin. Neurophysiol..

[19] Camarinha C.R., Frota S.M.M.C., Pacheco-Ferreira H., Lima M.A.M.T. (2011). Avaliação do processamento auditivo temporal em trabalhadores rurais expostos ocupacionalmente a agrotóxicos organofosforados. J. Soc. Bras. Fonoaudiol..

[20] Jayasinghe S.S., Pathirana K.D. (2011). Effects of deliberate ingestion of organophosphate or paraquat on brain stem auditory-evoked potentials. J. Med. Toxicol..

[21] Andrade M.I.K.P. (2012). Efeitos da Exposição ao Agrotóxico no Sistema Auditivo Eferente Através das Emissões Otoacústicas Transientes com Supressão. Ph.D. Thesis.

[22] Bazílio M.M.M., Frota S., Chrisman J.R., Meyer A., Asmus C.I.F., Camara V.M. (2012). Processamento auditivo temporal de trabalhadores rurais expostos a agrotóxicos. J. Soc. Bras. Fonoaudiol..

[23] Murthy V.A., Reddy I.J.V. (2012). Audiological assessment in organophosphorus compound poisoning. Indian J. Otolaryngol. Head Neck Surg..

[24] França D.M.V. (2013). Efeitos do uso dos Agrotóxicos no Sistema Auditivo Central dos Fumicultores da Região do Centro-Sul do Paraná. Ph.D. Thesis.

[25] Delecrode C.R. (2014). Processamento Auditivo em Trabalhadores Expostos a Ruído e Inseticidas: Testes de Ordenação Temporal e P300. Master’s Thesis.

[26] Alcarás P.A.S., Lacerda A.B.M., Marques J.M. (2013). Estudo das emissões otoacústicas evocadas e efeito de supressão em trabalhadores expostos a agrotóxicos e ruído. CoDAS.

[27] Lobato D.C.B. (2015). Disfunção Auditiva Induzida por Agrotóxicos em Trabalhadores Agrícolas do Paraná. Ph.D. Thesis.

[28] Guida H.L., Morini R.G., Cardoso A.C.V. (2010). Audiological evaluation in workers exposed to noise and pesticide. Braz. J. Otorhinolaryngol..

[29] Brasil (2008). Norma Regulamentadora 7–Programa de Controle Médico de Saúde Ocupacional. Portaria 19, Anexo I. Diretrizes e Parâmetros mínimos para avaliação e acompanhamento da audição em trabalhadores expostos a níveis de pressão sonora elevados. Manuais de Legislação-Segurança e Medicina do Trabalho.

[30] Vilela R.A.G., Malagoli M.E., Morrone L.C. (2005). Trabalhadores de risco: O uso de pulverizadores no controle de vetores. Production.

[31] Ministério da Saúde, Fundação Nacional de Saúde (2001). Controle de Vetores–Procedimento de Segurança–Manual do Supervisor de Campo.

[32] Pereira L.D., Schochat E. (1997). Processamento Auditivo Central–Manual de Avaliação.

[33] Collet L., Veuillet E., Bene J., Morgon A. (1992). Effects of contralateral white noise on click-evoked emissions in normal and sensorineural ears: Towards an exploration of the medial olivocochlear system. Audiology.

[34] Daniel W.W. (1995). Biostatistics: A Foundation for Analysis in the Health Sciences.

[35] Brasil, Ministério da Saúde (2019). Manual Sobre Medidas de Proteção à Saúde dos Agentes de Combate às Endemias. Arboviroses Transmitidas Pelo Aedes Aegypti.

[36] Sousa L.C.A., Piza M.R.T., Alvarenga K., Cóser P.L. (2016). Eletrofisiologia da Audição e Emissões Otoacústicas: Princípios e Aplicações Clínicas.

[37] Menezes P.L., Andrade K.C.L., Frizzo A.C.F., Carnaúba A.T.L., Lins O.G. (2018). Tratado de Eletrofisiologia Para Audiologia.

[38] Fuente A., McPherson B. (2007). Central auditory processing effects induced by solvent exposure. Int. J. Occup. Med. Environ. Health.

[39] Léonard M.R. (2011). Effet de la Co-Exposition au Bruit et aux Pesticides Organophosphorés sur l’audition des Travailleurs Agricoles. Master’s Thesis.

[40] Quevedo L.S., Tochetto T.M., Siqueira M.A. (2012). Condição coclear e do sistema olivococlear medial de frentistas de postos de gasolina expostos a solventes orgânicos. Arq. Int. Otorrinolaringol..

[41] Hood L.J., Berlin C.I. (1996). The role of otoacoustic emissions in identifying carriers of hereditary hearing loss. Otoacoustic Emissions Basic Science and Clinical Applications.

[42] Oliveira J.R.M., Fernandes J.C., Costa-Filho O. (2009). A Influência da idade na atividade do sistema eferente nas propriedades mecânicas da cóclea de ouvintes normais. Braz. J. Otorhinolaryngol..

